# Wigner Distribution Sets Universal Lower Bound for Quantum Advantage in Gaussian Boson Sampling

**DOI:** 10.3390/e28020188

**Published:** 2026-02-09

**Authors:** Vitaly V. Kocharovsky, Kunwar Kalra

**Affiliations:** 1 Department of Physics and Astronomy, Texas A&M University, College Station, TX 77843, USA; 2 Department of Mathematics, Texas A&M University, College Station, TX 77843, USA; kkalra1003@tamu.edu

**Keywords:** boson sampling, quantum advantage, computational complexity, Wigner distribution

## Abstract

The computational complexity, or quantum advantage, of Gaussian boson sampling is ascribed to squeezing of the Wigner quasiprobability distribution. This approach reveals the physical origin of the quantum complexity resource. This approach sets an easy-to-compute universal lower bound for the complexity dimension determined by the boson number in the quantum complexity resource. It is shown that the Wigner lower bound is close to the exact value of the complexity dimension obtained via numerical convex optimization. Our analytical and numerical results disclose a series of remarkable properties of quantum advantage.

## 1. Introduction: Physical Nature and Complexity Dimension of the Quantum Resource That Provides Quantum Advantage over Classical Computers

Analysis of the quantum computational advantage of many-body quantum systems over classical computers is one of the central topics of modern quantum information science [1,2,3,4,5,6,7,8]. Amazingly, even a relatively simple Gaussian mixed quantum state of a many-body boson system, such as a system of photons and atoms employed in photonic, atomic, or hybrid boson sampling [9,10,11,12,13,14,15], can demonstrate quantum advantage. The point is that quantum statistics of boson numbers in this case are given by matrix hafnians [13,16,17], which, in the general case, are ♯P-hard for computing. A matrix under hafnians in such a Gaussian system of a finite number *M* of boson modes is fully determined by its 2M×2M covariance matrix *V*.

Sampling is the generation of strings of numbers obeying a certain joint probability distribution. In the case of boson sampling, it is a measurement of numbers of quanta in boson modes. The intractability of computing the amplitudes of bosonic, linear-optical networks was associated with ♯P-complete permanents relatively recently [18,19], although permanents and hafnians had been introduced in quantum field theory long ago [20]. Boson sampling from a linear interferometer as a quantum simulator capable of demonstrating quantum advantage originally implied inputting photons into the pure Fock quantum states [8]. In the absence of the appropriate on-demand sources of Fock-state photons, only the subsequent invention of Gaussian boson sampling (GBS) [10,11,12], which admits optical parametric oscillators as the sources of input photons in squeezed-vacuum states, opened a path to advanced multimode experiments on boson sampling [21,22,23,24,25,26,27]. Boson sampling from nonlinear, interacting systems of bosons (atoms, photons, or coupled atoms and photons), suggested recently in [13,14,15,28] as an alternative to the usual linear interferometer systems, provides a new avenue for demonstrating quantum advantage that shows properties like those of GBS.

In real systems, the quantum ♯P-complexity is significantly suppressed by the presence of losses, including thermal and other sources of classical noise. This fact opens a path for efficient simulation of real quantum systems via classical algorithms. Specifically, the best of all known algorithms of this kind found in [29] allows for the simulation of ground-truth statistics of large-scale GBS experiments [21,22,23], performing better than the actual measurements in the experiments and requiring only about an hour of computing time. The algorithm is based on finding the system’s quantum complexity resource by means of numerical convex optimization and subsequent simulation of its hafnian-complex sampling by the tensor-network method of the matrix product state.

The essential computational complexity of GBS is set by the dimension $N_{q}$ of the matrix under these hafnians, which is equal to the boson number in the quantum resource given by the trace of the covariance matrix $V_{q}$ associated with the quantum complexity resource as follows: $N_{q}=\frac{1}{2}\mathrm{Tr}\left\{V_{q}-\frac{1}{2}\right\}$. It is named ‘*the complexity dimension of the quantum resource*’. (Do not mix this with the dimension 2M of the quantum resource’s covariance matrix or the full covariance matrix *V*, which is always twice the total number *M* of bosonic modes in the system.) Numerical simulations presented in [29] showed that losses and classical noise dramatically reduced the number $N_{q}$ of squeezed bosons constituting the quantum resource from the large input of 400 or 300 squeezed bosons in the large-scale GBS setups Borealis or Jiuzhang to just about 10 or 5 [21,22,23], respectively. Yet, physical origins of the squeezed bosons constituting the quantum complexity resource and the easy-to-compute estimate of their number were not disclosed in [29]: the quantum resource just appeared magically as an outcome of a powerful numerical convex optimization without explicitly pointing to the original bosons which actually formed this quantum complexity resource.

The present paper is devoted to the analysis of such Gaussian boson systems. We unveil the simple physical nature of the above quantum complexity resource and provide an easy-to-compute Wigner lower bound for the boson number in this resource, that is, the complexity dimension of the quantum resource and, hence, the entire GBS.

The idea is to trace the modes, which provide squeezed bosons to the quantum complexity resource, and the origin of the ♯P-hafnian-complex statistics of the discrete joint probability distribution of the sampled numbers of boson quanta directly from the overall quantum statistics of the system’s state, which is described by the density matrix operator $\hat{\rho }$ and fully represented by the Wigner quasiprobability distribution of the continuous coordinates and momenta of M bosonic modes. Wigner distribution is an easy-to-compute Gaussian distribution, and its geometrical complexity consists of deviation from spherical symmetry due to multimode squeezing in the multi-dimensional phase space. The ♯P-hard complexity of the Gaussian boson sampling appears in the course of reduction of the easy-to-compute continuous Wigner distribution to the discrete joint distribution of boson quanta in the same way as the ♯P-hard-for-computing hafnians of discrete variables appear in the course of Fourier series integration (transformation) of the easy-to-compute generating function of continuous variables in the Hafnian Master Theorem [13,16].

Thus, geometrical squeezing of the multi-dimensional ellipsoidal quasiprobability iso-density surfaces of the Wigner distribution along various minor axes is the reason for the existence of the nontrivial quantum complexity resource. The number of squeezed bosons in this quantum resource depends on the amount and direction of multivariate squeezing (the length and orientation of minor axes) of Wigner quasiprobability iso-density ellipsoids.

The content of this paper is as follows: In Section 2 we introduce the Wigner lower bound for quantum advantage. In Section 2.1 we propose a rigorous formula which defines the Wigner lower bound and explain the basic idea behind its definition. In Section 2.2 we prove that this formula indeed provides a universal lower bound for quantum complexity of the multimode boson system. Also, we present the exact explicit expression for the Wigner lower bound in the case when all modes have the same transmission coefficient and prove that in this case, the lower bound is equal to the exact value of the complexity dimension computed by means of convex optimization (semidefinite programming). Section 3 contains a detailed numerical analysis and comparison of the universal Wigner lower bound against the exact value given by numerical convex optimization for Gaussian boson sampling. In Section 3.1 we elaborate on the numerical protocol, sampling design, and diagnostic conventions. Section 3.2, Section 3.3, Section 3.4, Section 3.5 and Section 3.6 contain analyses of the dependence of Gaussian boson sampling quantum complexity on the interferometric unitaries, mode losses, mode squeezing, and number of modes. The spectrum of eigenvalues and spectral asymmetry of the covariance matrix are addressed in Section 3.7. The upper bound for quantum advantage is introduced and discussed in Section 4. The main results and conclusions are summarized in Section 5.

## 2. Lower Bound for the Complexity Dimension of the Quantum Resource: Loewner Order

Let us explicate the above idea. Consider the system of a finite number *M* of boson modes. Suppose the system is in a Gaussian state [7]. Its quantum statistical properties are described by a given statistical operator, that is, a density matrix operator $\hat{\rho }$.

### 2.1. The Basic Idea: The Boson Number in the Squeezed Vacuum Hidden in the Wigner Distribution

Instead of $\hat{\rho }$, we will employ the corresponding Wigner distribution *W* which provides a different, fully equivalent way to describe all quantum statistical properties of the system. Like any probability density function in the theory of probability, Wigner distribution is defined as a Fourier transform of the characteristic function χ(λ) of the statistical operator

(1) $$\chi \left(\lambda \right)=\mathrm{Tr}\left\{\hat{\rho }e^{{\bar{a}}^{†}\bar{\lambda }-{\bar{\lambda }}^{†}\bar{a}}\right\},\;W\left(\alpha \right)=\frac{1}{\pi^{2M}}\int \chi \left(\lambda \right)e^{{\bar{\lambda }}^{†}\bar{\alpha }-{\bar{\alpha }}^{†}\bar{\lambda }}d^{2}\bar{\lambda }.$$

Here, ${\bar{a}}^{†}=\left({\hat{a}}_{1}^{†},\ldots ,{\hat{a}}_{M}^{†}\right)$ is the row vector of the creation operators and $\bar{a}={\left({\hat{a}}_{1},\ldots ,{\hat{a}}_{M}\right)}^{T}$ is the column vector of the annihilation operators. The combined column of all 2M creation and annihilation operators is denoted as $\hat{a}={\left({\bar{a}}^{†},{\bar{a}}^{T}\right)}^{T}$. The superscript *T* denotes a transposition of a matrix. A Hermitian conjugation applied to a matrix of operators denotes a transposition of the matrix and a Hermitian conjugation of its entries. The column vector $\bar{\lambda }={\left(\lambda_{1},\ldots ,\lambda_{M}\right)}^{T}$ and the row vector ${\bar{\lambda }}^{†}=\left(\lambda_{1}^{*},\ldots ,\lambda_{M}^{*}\right)$ consist of the complex c-number displacement variables and their complex-conjugated counterparts, respectively; $\lambda ={\left(\lambda_{1}^{*},\ldots ,\lambda_{M}^{*},\lambda_{1},\ldots ,\lambda_{M}\right)}^{T}$ is the column vector of all displacement variables. The entries of the corresponding row and column vectors ${\bar{\alpha }}^{†}=\left(\alpha_{1}^{*},\ldots ,\alpha_{M}^{*}\right)$ and $\bar{\alpha }={\left(\alpha_{1},\ldots ,\alpha_{M}\right)}^{T}$ constitute the column $\alpha ={\left(\alpha_{1}^{*},\ldots ,\alpha_{M}^{*},\alpha_{1},\ldots ,\alpha_{M}\right)}^{T}$ of 2M complex variables of the Wigner distribution.

The physical meaning of the squeezed quantum state originates from a geometrical squeezing of the Wigner quasiprobability distribution in the phase space of coordinates and momenta $\left\{q_{j},p_{j}|j=1,\ldots ,M\right\}$ corresponding to the conjugated pairs of the coordinate and momentum operators of all *M* boson oscillators of the system obeying canonical commutation relations

(2) $${\hat{q}}_{j}=\frac{1}{\sqrt{2}}\left({\hat{a}}_{j}^{†}+{\hat{a}}_{j}\right),\;{\hat{p}}_{j}=\frac{i}{\sqrt{2}}\left({\hat{a}}_{j}^{†}-{\hat{a}}_{j}\right);\;\left[{\hat{a}}_{j},{\hat{a}}_{j^{\prime }}^{†}\right]=\delta_{jj^{\prime }},\;\left[{\hat{q}}_{j},{\hat{p}}_{j^{\prime }}\right]=i\delta_{jj^{\prime }}.$$

So, we will change variables in the Wigner distribution (1) by expressing displacement variables by their quadrature counterparts, $\alpha_{j}^{*}=\left(q_{j}-ip_{j}\right)/\sqrt{2},\alpha_{j}=\left(q_{j}+ip_{j}\right)/\sqrt{2}$, and will consider Wigner distribution as the function W(s) of M real-valued momenta $\left\{p_{j}\right\}$ and M real-valued coordinates $\left\{q_{j}\right\}$ constituting the 2M-column vector $s={\left(p_{1},\ldots ,p_{M},q_{1},\ldots ,q_{M}\right)}^{T}$.

At the same time, quantum statistics of the Gaussian system are fully described by its 2×2 block covariance matrix of momentum–coordinate or creation–annihilation operators:

(3) $$V=\left[\begin{array}{cc} \langle {\hat{p}}_{j}{\hat{p}}_{j^{\prime }}\rangle \; & \frac{1}{2}{\langle {\hat{x}}_{j}{\hat{p}}_{j^{\prime }}+{\hat{p}}_{j^{\prime }}{\hat{x}}_{j}\rangle }^{T}\\ \frac{1}{2}\langle {\hat{x}}_{j}{\hat{p}}_{j^{\prime }}+{\hat{p}}_{j^{\prime }}{\hat{x}}_{j}\rangle \; & \langle {\hat{x}}_{j}{\hat{x}}_{j^{\prime }}\rangle \end{array}\right]=\langle \hat{s}{\hat{s}}^{T}\rangle +\frac{i}{2}\Omega ,\;G=\left[\begin{array}{cc} \langle {\hat{a}}_{j}^{†}{\hat{a}}_{j^{\prime }}\rangle \; & \langle {\hat{a}}_{j}^{†}{\hat{a}}_{j^{\prime }}^{†}\rangle \\ \langle {\hat{a}}_{j}{\hat{a}}_{j^{\prime }}\rangle \; & \langle {\hat{a}}_{j}^{†}{\hat{a}}_{j^{\prime }}\rangle \end{array}\right].$$

Here, the angular brackets denote an average (a trace) over the quantum ensemble described by the density matrix operator $\hat{\rho },\langle \ldots \rangle =\mathrm{Tr}\;$$\left\{\ldots \hat{\rho }\right\}$. The matrix Ω is the canonical symplectic matrix, and the two covariance matrices are related by the unitary transformation:

(4) $$\Omega =\left[\begin{array}{cc} 0\; & I_{M}\\ -I_{M}\; & 0 \end{array}\right],\;G+\frac{1}{2}I_{2M}=\tau V\tau^{†},\;\tau =\frac{1}{\sqrt{2}}\left[\begin{array}{cc} -iI_{M}\; & I_{M}\\ iI_{M}\; & I_{M} \end{array}\right],\;\hat{a}=\tau \hat{s},$$

where the 2×2 block unitary τ relates the 2M-column vector of creation–annihilation operators $\hat{a}$ to the 2M-column vector of momenta–coordinate operators $\hat{s}={\left({\hat{p}}_{1},\ldots ,{\hat{p}}_{M},{\hat{q}}_{1},\ldots ,{\hat{q}}_{M}\right)}^{T}$.

Our analysis is based on the well-known fact [7] that the Wigner distribution for the Gaussian state is equal to the Gaussian distribution determined by the covariance matrix,

(5) $$W\left(s\right)=\frac{1}{{\left(2\pi \right)}^{M}\sqrt{\det V}}\exp\left(-\frac{1}{2}s^{T}V^{-1}s\right),$$

where we assume that it is centered at the origin of the phase space.

The covariance matrix *V* for any physical system is symmetric and positive definite. All of its eigenvalues are positive $\lambda_{j}>0,j=1,\ldots ,2M$, and their reciprocals determine the major and minor axes of the 2M-dimensional probability iso-density ellipsoids of the Wigner distribution, which is non-negative for any Gaussian state [7].

Our basic idea is to ascribe quantum computational complexity to the deviation of the Wigner quasiprobability iso-density ellipsoids from the symmetrical spheres caused by nonclassical squeezing associated with the eigenvalues less than the vacuuam threshold $\lambda_{\mathrm{vac}}=\frac{1}{2}$, $\left\{\lambda_{j}^{\left(-\right)}\in \left(0,\frac{1}{2}\right)|j=1,\ldots ,K\right\}$. Here, *K* is the number of such eigenvalues of the quadrature covariance matrix *V*; K≤M. We mark such eigenvalues with a superscript ‘(−)’ since they provide negative eigenvalues $\lambda_{j}^{\left(-\right)}-1/2<0$ for the covariance matrix *G* of the creation and annihilation operators, as per Equations (3) and (4). Every eigenvector $s^{\left(j\right)}$ of the quadrature covariance matrix *V* with such an eigenvalue $\lambda_{j}^{\left(-\right)}<\lambda_{\mathrm{vac}}=1/2$ determines a direction in the phase space that corresponds to a superposition of coordinates and momenta of the bare M boson modes and can be associated with the coordinate or momentum operators of some mixed boson mode. Let us construct the quantum computational resource of the original system as a system of all such mixed superposition modes orthogonalized by an appropriate symplectic Gram–Schmidt procedure [30] and assign each of them to be in the pure vacuum-squeezed state with the corresponding single-mode squeezing parameter $r_{j}$ and boson number $n_{j}=\sinh^{2}r_{j}$, shown as follows:

(6) $$\lambda_{j}^{\left(-\right)}=\left(1/2\right)\exp\left(-2r_{j}\right),\;n_{j}=\sinh^{2}r_{j}=\left[2\lambda_{j}^{\left(-\right)}-2+1/\left(2\lambda_{j}^{\left(-\right)}\right)\right]/4,\;j=1,\ldots ,K.$$

The quantum advantage of the lossy GBS and other many-body boson systems over classical computers is associated with the computational complexity of classical-algorithm simulation of the squeezed multi-dimensional Wigner quasiprobability distribution as compared to the spherically symmetric distribution and can be characterized by the total boson number in the above superposition modes being in the squeezed-vacuum state,

(7) $$N_{W}=\sum_{j=1}^{K}\sinh^{2}r_{j}=\frac{1}{4}\sum_{\lambda_{j}^{\left(-\right)}<1/2}\left(2\lambda_{j}^{\left(-\right)}-2+\frac{1}{2\lambda_{j}^{\left(-\right)}}\right).$$

We call this boson number $N_{W}$ *the Wigner lower bound*, since we prove below that it gives the lower bound for the dimension of the matrix which determines the sampling probability distribution via the ♯P-complex hafnian and directly points to the superposition modes which constitute the quantum complexity resource hidden under the classically simulatable fluctuations/noise but are encrypted into the density matrix operator which is fully described by the Wigner distribution.

Let us compare the quantum computational resource, built by the above deterministic constructive procedure via the standard spectral decomposition of the covariance matrix *V*, against the quantum resource obtained by numerical convex optimization in [29]. The approach in [29] consists of the decomposition of the covariance matrix, $V=V_{q}+V_{c}$, into the sum of the quantum resource part $V_{q}$ and classical part $V_{c}$. The quantum part $V_{q}$ corresponds to a direct sum of squeezed-vacuum states and requires a hafnian-complex, potentially ♯P-hard computing by the tensor-network method. The classical part is a positive semidefinite matrix, $V_{c}⪰0$, and can be interpreted as a Gaussian random displacement which is easy to simulate classically. The algorithm in [29] implements the optimized decomposition procedure by using semidefinite programming under the constraints:

(8) $$\min_{V_{q}}\mathrm{Tr}\left\{V_{q}\right\}\;\mathrm{with}\;V-V_{q}⪰0,\;V_{q}⪰\frac{i}{2}\Omega ⊗I_{M};\;V=V_{q}+V_{c}.$$

Here, the first constraint is to ensure that $V_{c}=V-V_{q}$ is positive semidefinite. The second constraint is to guarantee that $V_{q}$ represents a proper physical Gaussian state. The minimization is to minimize the quantum part’s average boson number. After numerically computing the matrix $V_{q}$ and obtaining all of its eigenvalues in ascending order, the algorithm in [29] calculates the dimension of computational complexity as the total boson number in the quantum resource $V_{q}$ via a formula similar to Equation (7),

(9) $$N_{q}=\frac{1}{4}\sum_{j=1}^{M}\left(2\mu_{j}-2+\frac{1}{2\mu_{j}}\right)=\frac{1}{2}\mathrm{Tr}\left\{V_{q}-\frac{1}{2}I_{2M}\right\};\;\mu_{1}\leq \mu_{2}\leq \cdots \leq \mu_{2M}.$$

Below we compare, analytically and numerically, the result for the GBS computational complexity obtained by means of our simple estimate, Equation (7), based on the nonclassical eigenvalues of the total covariance matrix *V*, against the exact numerical result computed by means of finding the quantum resource’s covariance matrix $V_{q}$ via a blind numerical convex optimization and the summation of its eigenvalues or calculation of its trace in Equation (9).

### 2.2. Proof of Wigner Universal Lower Bound for Quantum Complexity of the Covariance Matrix

An arbitrary 2M×2M covariance matrix *V* is a symmetric positive definite matrix with real-valued entries. It can be diagonalized by an orthogonal transformation as follows

(10) $$V=Q\Lambda Q^{T},\;\Lambda =\mathrm{diag}\left\{\lambda_{j}|j=1,\ldots ,2M\right\};\;\lambda_{1}\leq \cdots \leq \lambda_{K}<\frac{1}{2}\leq \lambda_{K+1}\leq \cdots \leq \lambda_{2M}.$$

Here, the diagonal matrix Λ consists of 2M positive eigenvalues which we enumerate (including possible degeneracies) in ascending order. The first *K* of them are below the nonclassical threshold, that is, less than 1/2; the remaining 2M−K of them are larger or equal 1/2. *Q* is an orthogonal matrix whose columns are the real, orthonormal eigenvectors of *V*.

In the case of one boson mode, M=1, it is straightforward to prove analytically that our estimate in Equation (7) always gives the exact result:

(11) $$N_{W}=N_{q}\;\mathrm{if}\;M=1.$$

In fact, the 2×2 covariance matrix *V* can be decomposed into quantum and classical parts of Equation (8) explicitly at the level of the diagonal representation Λ in Equation (10) if we associate the first and second eigenvectors of *V* with the coordinate and momentum operators of the new mode, properly renormalized by the rotation in the phase space determined by the orthogonal matrix *Q* in accordance with the bare-mode correlations $V_{jj^{\prime }}$,

(12) $$V=\left[\begin{array}{cc} V_{11}\; & V_{12}\\ V_{12}\; & V_{22} \end{array}\right]=Q\left[\begin{array}{cc} \lambda_{1}\; & 0\\ 0\; & \lambda_{2} \end{array}\right]Q^{T}=Q\left[\begin{array}{cc} \frac{1}{2}e^{-2r_{1}}\; & 0\\ 0\; & \frac{1}{2}e^{2r_{1}} \end{array}\right]Q^{T}+Q\left[\begin{array}{cc} 0\; & 0\\ 0\; & \xi \end{array}\right]Q^{T}=V_{q}+V_{c}.$$

The exact quantum resource’s part $V_{q}$ is reached if we associate it with the correlations of the above renormalized mode in the squeezed-vacuum state with the single-mode squeezing parameter $r_{1}$ providing the entire value of the first eigenvalue and some part of the second eigenvalue:

(13) $$\lambda_{1}=\frac{1}{2}e^{-2r_{1}}\leq \frac{1}{2},\;\lambda_{2}=\frac{1}{2}e^{2r_{1}}+\xi \geq \frac{1}{2}.$$

In this way, we exactly nullify the first eigenvalue in the classical part $V_{c}$. The remaining non-negative part ξ≥0 of the second eigenvalue in $V_{c}$ is responsible for the fluctuations which can be simulated classically via sampling displacements with the Gaussian distribution. Obviously, this choice of $V_{q}$ provides the exact maximum of the nonclassical quantum complexity one can hide under classical noise since further increases in the squeezing parameter $r_{1}$ would make the first eigenvalue of the classical part $V_{c}$ negative, that is, would make $V_{c}$ nonpositive definite, preventing its classical simulation. The above construction in Equation (12) and the irreducible boson number, $\sinh^{2}r_{1}$, which remains in the quantum computational resource $V_{q}$ and measures the complexity dimension, are exactly the same as prescribed by our estimate in Equation (7).

The above proof of the exact equality in Equation (11) is valid for an arbitrary state of the boson mode, both at an entrance to a lossy interferometer where the eigenvalues of the quadrature covariance matrix are $\lambda_{1,2}^{\left(in\right)}$ and at the interferometer’s output, after the mode decays in power by the transmission factor η<1 and acquires the renormalized eigenvalues $\lambda_{1,2}^{\left(out\right)}$. In the latter case, the eigenvalues of the covariance matrix *G* at the output of such a lossy single-mode interferometer scale with the factor η, which yields

(14) $$\lambda_{1,2}^{\left(out\right)}=\eta \left(\lambda_{1,2}^{\left(in\right)}-\frac{1}{2}\right)+\frac{1}{2}=\eta \lambda_{1,2}^{\left(in\right)}+\frac{1-\eta }{2}$$

and the exact result for the number of photons in the quantum resource, determined by Equation (7), at output

(15) $$N_{W}^{\left(out\right)}=N_{q}^{\left(out\right)}=\frac{\eta^{2}{\left(\lambda_{1}^{\left(in\right)}-1/2\right)}^{2}}{2\eta (\lambda_{1}^{\left(in\right)}-1/2)+1}\;,\;M=1.$$

In the case of a pure squeezed-vacuum input state, when $\lambda_{1,2}^{\left(in\right)}=\left(1/2\right)e^{\mp 2r}$, we have

(16) $$\lambda_{1,2}^{\left(out\right)}=\eta \frac{e^{\mp 2r}}{2}+\frac{1-\eta }{2},\;N_{W}^{\left(out\right)}=N_{q}^{\left(out\right)}=\frac{\eta^{2}{\left(e^{-2r}-1\right)}^{2}}{4[\eta \left(e^{-2r}-1\right)+1]}\;,\;M=1.$$

For the multimode lossy interferometer, M≥2, the equality of the lower bound (7) and the exact value (9) for the complexity dimension, $N_{W}^{\left(out\right)}=N_{q}^{\left(out\right)}$, is valid for an arbitrary transmission coefficient η<1 only if losses are the same for all modes, $\eta_{j}=\eta =\mathrm{const}$. In this case, the covariance matrix *G* and all its eigenvalues scale homogeneously with the factor η, like in Equation (14), since a two-block unitary mixing between modes in the interferometer leaves invariant the canonical commutation relation between coordinate and momentum operators, rotating them in each conjugated pair synchronously. Therefore, due to losses, each mode scales independently on the other modes, following exactly the same pattern as in the single-mode case, as shown in Equations (14)–(16). The result for the multimode complexity dimension is equal to the sum over all single-mode contributions in Equation (15) or Equation (16), even if the state parameters (eigenvalues $\lambda_{j}^{\left(in\right)}$; squeezing parameters $r_{j}$) are different for different modes:

(17) $$N_{W}^{\left(out\right)}=N_{q}^{\left(out\right)}=\sum_{\lambda_{j}^{\left(in\right)}<1/2}\frac{\eta^{2}{\left(\lambda_{j}^{\left(in\right)}-1/2\right)}^{2}}{2\eta (\lambda_{j}^{\left(in\right)}-1/2)+1}\;,\;\eta_{j}=\eta =\mathrm{const},\;M\geq 1.$$

In the case of squeezed-vacuum input states with arbitrary squeezing parameters, we have

(18) $$N_{W}^{\left(out\right)}=N_{q}^{\left(out\right)}=\frac{1}{4}\sum_{r_{j}}\frac{\eta^{2}{\left(e^{-2r_{j}}-1\right)}^{2}}{\eta (e^{-2r_{j}}-1)+1}\;,\;\;\lambda_{j}^{\left(in\right)}=\frac{e^{-2r_{j}}}{2},\;\;\eta_{j}=\eta =\mathrm{const},\;M\geq 1.$$

If there are two or more boson modes, M≥2, and if losses are not the same for all modes, then usually the lower bound does not coincide with the exact result. Remarkably, even in the general case, it is easy to prove that our estimate of the complexity dimension in Equation (7) gives the exact lower bound for quantum complexity of the covariance matrix. This means that for arbitrary parameters of the multimode boson system, one always has

(19) $$N_{W}\leq N_{q}.$$

The proof of the exact inequality in Equation (19) in the general case immediately follows from the Loewner order for two real symmetric matrices whose difference is semidefinite positive by constructing Equation (8), $V_{c}=V-V_{q}⪰0$, which entails the same order (inequality) for each pair of corresponding eigenvalues of these two matrices. Assuming that the eigenvalues are enumerated in the ascending order as above, we have

(20) $$V⪰V_{q}\;\Rightarrow \;\lambda_{j}\geq \mu_{j},\;j=1,\ldots ,2M.$$

The implication in Equation (20) follows from the min-max theorem that provides the eigenvalues of the matrices *V* and $V_{q}$ for any j∈{1,…,2M}, as follows

(21) $$\lambda_{j}=\min_{L\subset C^{2M},\;\dim\left(L\right)=j}\;\max_{x\in L\setminus \{0\}}\frac{x^{T}Vx}{x^{T}x}\;\geq \;\mu_{j}=\min_{L\subset C^{2M},\;\dim\left(L\right)=j}\;\max_{x\in L\setminus \{0\}}\frac{x^{T}V_{q}x}{x^{T}x}.$$

The inequality in Equation (21) is due to $V⪰V_{q}$, which is the same as $x^{T}Vx\geq x^{T}V_{q}x$.

For the corresponding squeezing parameters defined in Equations (6)–(9) for the first *K* eigenvalues via equations $\lambda_{j}=\left(1/2\right)e^{-2r_{j}},\mu_{j}=\left(1/2\right)e^{-2r_{j}^{\left(q\right)}},j=1,\ldots ,M$, we have the opposite inequalities, which are also valid for the numbers of bosons in each j-th squeezed-vacuum mode, contributing to the total boson number in the quantum resources introduced on the basis of Wigner distribution, Equation (7), and convex optimization, Equation (9):

(22) $$r_{j}\leq r_{j}^{\left(q\right)}\;\Rightarrow \;n_{j}=\sinh^{2}r_{j}\leq n_{j}^{\left(q\right)}=\sinh^{2}r_{j}^{\left(q\right)}\;\forall j=1,\ldots ,K\;\Rightarrow \;N_{W}\leq N_{q}.$$

## 3. Quantum Complexity of Gaussian Boson Sampling: Numerical Convex Optimization vs. Universal Wigner Lower Bound

To simplify interpretation of the subsequent numerical analysis, we employ a basic physical model of lossy Gaussian boson sampling with the ground-truth covariance matrix of a special kind. Let us assume that at the entrance of an interferometer there is some number, say M=1,…,30, of boson modes, each of which is in a squeezed-vacuum state with a single-mode squeezing parameter $r_{j},j=1,\ldots ,M,$ chosen from the interval $r_{j}\in \left[0.05,2.5\right]$. The input covariance matrices for the coordinate and momentum operators, $V^{\left(in\right)}$, and for the creation and annihilation operators, $G^{\left(in\right)}$, are given in line with Equations (3) and (4) as follows

(23) $$V^{\left(in\right)}=\mathrm{diag}\left\{\lambda_{j}^{\left(in\right)}\right\}=V_{q}^{\left(in\right)}=\mathrm{diag}\left\{\mu_{j}^{\left(in\right)}\right\},\;G^{\left(in\right)}=\tau V^{\left(in\right)}\tau^{†}-\frac{1}{2}I_{2M}\;.$$

The input eigenvalues come in pairs, and eigenvalues of the entire quadrature covariance matrix *V* coincide with the eigenvalues of the quantum resource’s part $V_{q}$:

(24) $$\lambda_{j}^{\left(in\right)}=\mu_{j}^{\left(in\right)}=\frac{e^{-2r_{j}}}{2}<\frac{1}{2}\;,\;\lambda_{M+j}^{\left(in\right)}=\mu_{M+j}^{\left(in\right)}=\frac{e^{2r_{j}}}{2}>\frac{1}{2}\;;\;j=1,\ldots ,M.$$

So, at the interferometer’s entrance, classical noise is absent, $V_{c}^{\left(in\right)}=0$, and all input modes belong to the quantum complexity resource, $V^{\left(in\right)}=V_{q}^{\left(in\right)}$. All input modes are squeezed below the nonclassical threshold, $\lambda_{j}<\lambda_{\mathrm{vac}}=1/2,j=1,\ldots ,M$, and K=M in Equations (6) and (10).

Let us assume that the input squeezed light propagates through a mixing ideal interferometer described by a 2M×2M two-block-diagonal unitary ${\tilde{U}}_{1}$, then each mode experiences attenuation due to losses on beam splitters described by the amplitude, $t_{j}$, and power, $\eta_{j}=t_{j}^{2}\in \left[0.01,1\right]$, transmission coefficients, and finally light passes through the second interferometer described by a 2M×2M two-block-diagonal unitary ${\tilde{U}}_{2}$. The M×M unitaries $U_{1}$ and $U_{2}$ are chosen randomly. So, the covariance matrix for the creation and annihilation operators at the output of the lossy interferometer is given by the following transformation:

(25) $$G=TG^{\left(in\right)}T^{†},\;T={\tilde{U}}_{1}D{\tilde{U}}_{2};\;{\tilde{U}}_{1,2}=\left[\begin{array}{cc} U_{1,2}^{*} & 0\\ 0 & U_{1,2} \end{array}\right],\;D=\mathrm{diag}\left\{t_{j}|j=1,\ldots ,2M\right\},\;t_{j}=t_{M+j}.$$

Such a lossy transformation generates classical noise (that is, creates a nonzero classical part $V_{c}≻0$ of the covariance matrix *V*) and suppresses the computational complexity of the quantum resource’s part $V_{q}$ in Equation (8) at the output of the interferometer, since scaling factors $\eta_{j}<1$ significantly change covariance matrix eigenvalues. However, in the general case of a non-singular transfer matrix *T*, Sylvester’s law of inertia ensures that the number of negative, zero, and positive eigenvalues of the covariance matrix *G* remain the same. This means that the output number *K* of eigenvalues of the covariance matrix *V* which are below the nonclassical threshold, $\lambda_{j}<\lambda_{\mathrm{vac}}=1/2$ (see Equations (6) and (10)), remains the same as it was at the input, that is, K=M, as per the above choice of parameters in Equation (24).

### 3.1. Numerical Protocol, Sampling Design, and Diagnostic Conventions

Following the notations introduced in Section 2.1, we work in a convention where the 2M×2M vacuum quadrature covariance matrix for the system of *M* modes is $V_{\mathrm{vac}}=\frac{1}{2}I_{2M}$ and the vacuum variance threshold for eigenvalues is $\lambda_{\mathrm{vac}}=\frac{1}{2}$. All photon-number proxies below are defined relative to this convention.

#### 3.1.1. Forward Model Implemented in the Code

For each instance, we generate mode-wise squeezing parameters $r_{j}\in \left[0.05,2.5\right]$ and mode-wise *amplitude* transmissions $t_{j}\in \left[0.01,1\right],j=1,\ldots ,M$; assemble the complex-basis covariance matrix *G*; and then convert to the quadrature covariance matrix $V=\tau^{†}\;\left(G+\frac{1}{2}I_{2M}\right)\;\tau$, as per the notations in Equations (3) and (4). Loss is implemented by a diagonal transmission matrix in the complex basis, and the overall lossy transformation is given in Equation (25). In the implementation, the two unitaries $U_{1},U_{2}$ are sampled independently from the Haar measure on U(M) and lifted to ${\tilde{U}}_{1,2}$ in the complex basis.

#### 3.1.2. Physicality Checks and Numerical Tolerances

For each realized quadrature covariance *V*, we verify the Heisenberg condition,

(26) $$V+\frac{i}{2}\Omega ⪰0,$$

up to a numerical tolerance. In the recorded diagnostics, we also store minimum eigenvalues of *V* and semidefinite programming (SDP) feasibility blocks. We adopt a *violation tolerance* $\delta_{\mathrm{viol}}$ and treat deviations smaller than $\delta_{\mathrm{viol}}$ as numerical noise (in our runs $\delta_{\mathrm{viol}}=10^{-6}$).

#### 3.1.3. Quantum-Resource Boson Number from Euclidean Eigenvalues of Wigner Distribution

Let ${\left\{\lambda_{j}\right\}}_{j=1}^{2M}$ denote the (ordinary) eigenvalues of the symmetric covariance matrix *V*, sorted in ascending order. Each eigenvalue $\lambda_{j}<\lambda_{\mathrm{vac}}=\frac{1}{2}$ corresponds to sub-vacuum, nonclassical fluctuations along some orthogonal principal axis of Wigner quasiprobability iso-density ellipsoids and defines the associated effective squeezing $r_{j}$ and boson number contribution $n_{j}=\sinh^{2}\left(r_{j}\right)$ to the total boson number $N_{W}$ constituting the lower bound for the complexity dimension calculated on the basis of the Wigner distribution as per Equations (6) and (7). In the implementation, we guard against near-threshold numerical instabilities by using a small cutoff $\varepsilon_{W}$ (default $10^{-9}$) when testing $\lambda_{j}<1/2$.

#### 3.1.4. Semidefinite Programming (SDP) Decomposition Solved in the Implementation

Given the covariance matrix *V*, the SDP variable is a symmetric matrix $V_{q}⪰0$ intended to represent the minimal quantum resource part. The optimization solved is

(27) $$\begin{array}{cc} \min_{V_{q}}\; & \mathrm{Tr}(V_{q}) \end{array}$$

(28) $$\begin{array}{cc} \mathrm{subject}\;\mathrm{to}\;\; & \left(\begin{array}{cc} V_{q} & \Omega /2\\ -\Omega /2 & V_{q} \end{array}\right)⪰-\varepsilon_{\mathrm{SDP}}\;I_{4M}, \end{array}$$

(29) $$\begin{array}{cc} \; & V-V_{q}⪰-\varepsilon_{\mathrm{SDP}}\;I_{2M}, \end{array}$$

(30) $$\begin{array}{cc} \; & V_{q}⪰-\varepsilon_{\mathrm{SDP}}\;I_{2M}, \end{array}$$

where $\varepsilon_{\mathrm{SDP}}\geq 0$ is a small relaxation parameter used only to stabilize marginal instances. We emphasize that Equation (28) is the Heisenberg condition written as a real LMI block, and Equations (29) and (30) enforce the positive semidefiniteness of the decomposition. The SDP boson number proxy $N_{q}$ used in the code is the standard trace-based formula in Equation (9).

#### 3.1.5. Primary Tightness Metrics and Sign Conventions

We report two complementary tightness diagnostics:

(31) $$\rho \;=\;\frac{N_{W}}{N_{q}},\;\Delta \;=\;N_{q}-N_{W}.$$

Our analytic claim is ρ≤1, or equivalently Δ≤0 instance-wise. Because both quantities are computed numerically (eigen-decompositions and SDP solves), extremely small apparent “violations” of size $O\;(10^{-9})$ can occur and are treated as numerical tolerance effects.

#### 3.1.6. Transmission Metrics and Heterogeneity Statistics

Each mode has an amplitude transmission $t_{j}\in \left[0,1\right]$ and power transmission $\eta_{j}=t_{j}^{2}$. The code supports sampling and enforcing target means in one of three metrics:

(32) $$\mathrm{transmission}:\;t_{j},\;\mathrm{power}\_\mathrm{transmission}:\;\eta_{k}=t_{j}^{2},\;\mathrm{loss}\_\mathrm{fraction}:\;\ell_{j}=1-\eta_{j}.$$

When enforcing a target mean in a chosen metric, the profile is constructed in that metric, mean-corrected, and then mapped back to amplitude transmissions. Loss heterogeneity is quantified by the coefficient of variation

(33) $$\mathrm{CV}\left(\eta \right)\;=\;\frac{\mathrm{StandardDeviation}(\eta_{1},\ldots ,\eta_{M})}{\mathrm{Mean}(\eta_{1},\ldots ,\eta_{M})},$$

and analogously for squeezing heterogeneity CV(r).

#### 3.1.7. Additive vs. Multiplicative Profile Noise

Let $x_{k}$ denote a bounded per-mode profile parameter in the range $\left[x_{\min},x_{\max}\right]$, either a squeezing entry $r_{k}$ in $\left[r_{\min},r_{\max}\right]$ or a transmission metric variable $\eta_{k}$ in $\left[\eta_{\min},\eta_{\max}\right]$. The implementation supports two noise models controlled by a dimensionless noise fraction $\sigma_{\mathrm{prof}}$.

Additive noise:

(34) $$x_{k}\;=\;{\mathrm{clip}}_{\left[x_{\min},x_{\max}\right]}\;\left(x_{k}^{\left(0\right)}+\epsilon_{k}\right),\;\epsilon_{k}∼N\;\left(0,\;{\left(\sigma_{\mathrm{prof}}\left(x_{\max}-x_{\min}\right)\right)}^{2}\right),$$

so the additive noise scale is proportional to the allowed span.

Multiplicative noise:

(35) $$x_{k}\;=\;{\mathrm{clip}}_{\left[x_{\min},x_{\max}\right]}\;\left(x_{k}^{\left(0\right)}\;e^{\sigma_{\mathrm{prof}}\xi_{k}}\right),\;\xi_{k}∼N\left(0,1\right),$$

which is a lognormal multiplicative factor with a median unit. At fixed nominal $\sigma_{\mathrm{prof}}$, multiplicative noise typically induces smaller CV after clipping and target-mean enforcement, which empirically yields tighter bounds.

#### 3.1.8. Target-Mean Enforcement

When we require a target mean $\bar{x}$ (in the chosen metric), after applying noise, we perform a mean correction

(36) $$x_{k}\leftarrow {\mathrm{clip}}_{\left[x_{\min},x_{\max}\right]}\;\left(x_{k}+\delta \right),\;\delta =\bar{x}-\frac{1}{M}\sum_{k=1}^{M}x_{k},$$

and (optionally) iterate this correction a small number of times to compensate for mean drift introduced by clipping. This design isolates the heterogeneity effects at fixed mean transmission or fixed mean squeezing, depending on the sweep.

#### 3.1.9. Sampling Modes

We use two sampling schemes. In per_mode_uniform, each per-mode parameter is sampled and i.i.d. uniformly on the configured range (possibly in a chosen transmission metric), producing broad, physically diverse instances. In instance_mean_stratified, target means are sampled uniformly over a set of mean bins in the chosen variable(s), and then per-mode profiles are drawn with noise and mean enforcement; this yields approximately uniform coverage in $\left(\bar{r},\bar{\eta }\right)$ while preserving controlled heterogeneity at each mean point.

### 3.2. Fluctuations in GBS Quantum Computational Complexity Within Large Ensembles of Unitaries

#### 3.2.1. Validation of the Analytical Solution in Equation (18) and Exact Equality $N_{W}=N_{q}$ in the Case of the Homogeneous Loss Profile $\{\eta_{j}\equiv \bar{\eta }=\mathrm{const}\;|j=1,\ldots ,M$}

We first validate the implementation in the homogeneous regime, where the Wigner lower bound $N_{W}$ coincides with the exact value $N_{q}$ instance-wise as per Equation (18). We fix M=30, mean squeezing $\bar{r}=0.8$, and mean power transmission $\bar{\eta }=0.3$, with homogeneous profiles (no profile noise), and only vary the Haar-random interferometer. For each of n=1000 instances, we compute $N_{W}\left(V\right)$ from Equation (7) and $N_{q}\left(V_{q}\right)$ from Equation (9), and we report boson numbers $N_{W}$ and $N_{q}$ as well as their ratio ρ from Equation (31). As expected, we obtain $N_{W}=N_{q}$ and ρ=1 in all instances, including instances with different values of transmission η=const and the presence of arbitrary variation in squeezing over the modes.

#### 3.2.2. General Case of a Heterogeneous Loss Profile $\{\eta_{j}\neq \mathrm{const}\;|j=1,\ldots ,M$}

**Figure 1:**
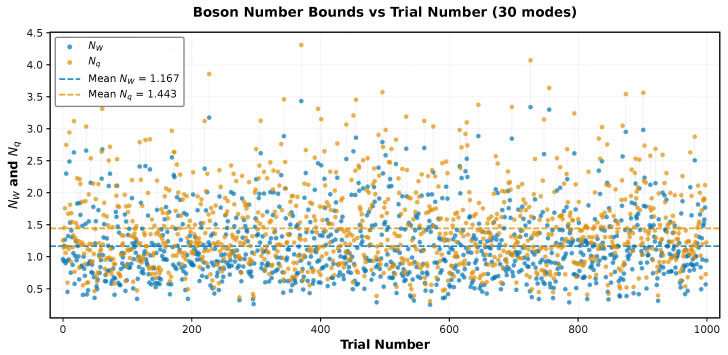
The Wigner lower bound, $N_{W}$, and the exact convex-optimization value, $N_{q}$, of the number of squeezed bosons in the quantum resource of the system of M=30 boson modes for 1000 instances of Haar-random unitaries, responsible for the interferometric mixing of modes, and random sets of transmission coefficients $\eta_{j}\in \left[0,1\right]$ and squeezing parameters $r_{j}\in \left[0.05,2.5\right],j=1,\ldots ,M$. $N_{W}$ and $N_{q}$ experience very large (about an order of magnitude) but always synchronous fluctuations.

**Figure 2:**
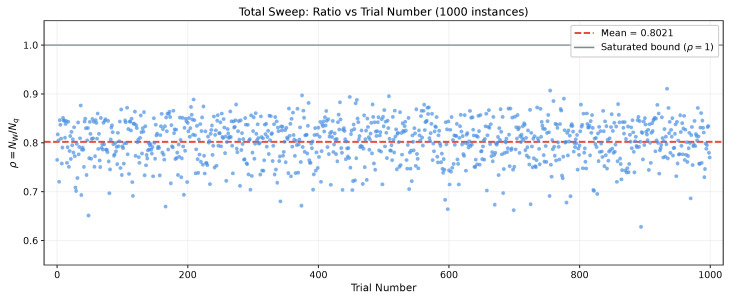
The ratio $\rho =N_{W}/N_{q}$ of the Wigner lower bound, $N_{W}$, and the exact convex-optimization value, $N_{q}$, of the number of squeezed bosons in the quantum resource of the system of M=30 boson modes for 1000 instances of Haar-random unitaries, responsible for the interferometric mixing of modes, and random sets of transmission coefficients $\eta_{j}\in \left[0,1\right]$ and squeezing parameters $r_{j}\in \left[0.05,2.5\right]$, j=1, …,M. Note that the lower bound estimates the exact value of the complexity dimension with a good accuracy of ∼20%, and the ratio fluctuates around its mean value $\bar{\rho }\approx 0.8$ only by about 10%, while fluctuations of boson numbers $N_{W}$ and $N_{q}$ are very large, about an order of magnitude, as per Figure 1.

Next, for the same average parameters, we introduce profile noise (heterogeneous profiles) for both losses and squeezing with the dimensionless noise fraction $\sigma_{\mathrm{prof}}=0.3$. For each of n=1000 instances we compute $N_{W}\left(V\right)$ from Equation (7) and $N_{q}\left(V_{q}\right)$ from Equation (9), and we report the quantum resource’s boson numbers $N_{W}$ and $N_{q}$ in Figure 1 as well as their ratio ρ from Equation (31) in Figure 2. From Figure 1, we conclude that both boson numbers $N_{W}$ and $N_{q}$ greatly depend on the choice of Haar unitary in the interferometric mixing and fluctuate by an order of magnitude in the range of about 0.4 to 4. Remarkably, they fluctuate (jump) from instance to instance simultaneously, remaining relatively close to each other and always in the proper order $N_{W}\leq N_{q}$. Thus, the total sweep of 1000 instances of the ratio $\rho =N_{W}/N_{q}$ shown in Figure 2 clearly confirms that the Wigner boson number $N_{W}$ in Equation (7) is indeed the lower bound for the complexity dimension and closely represents the exact complexity dimension $N_{q}$ with an accuracy of about 20% for typical parameters of GBS experiments.

### 3.3. Dependence of GBS Quantum Complexity on the Mode Losses

This section characterizes how quantum complexity and tightness of the Wigner lower bound depend on the mean value and variance in losses across the modes of the system.

**Figure 3:**
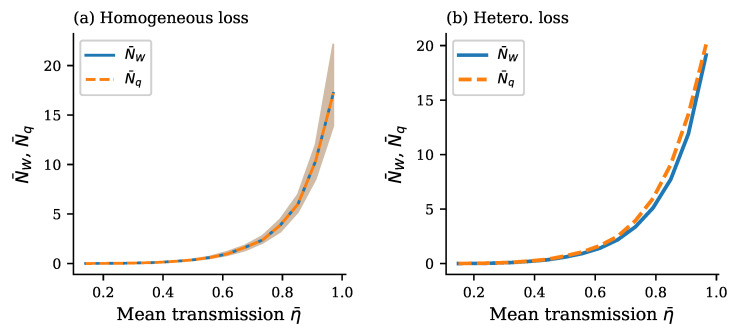
Mean values of the Wigner lower bound ${\bar{N}}_{W}$ (solid blue curve) and the exact convex optimization ${\bar{N}}_{q}$ (dashed orange curve) of boson numbers in the quantum resource (complexity dimension) as functions of the mean transmission $\bar{\eta }$ at constant squeezing r=0.8 for the system of M=30 boson modes. (**a**) *Homogeneous loss profile:* When all modes experience identical transmission $\eta_{j}=\bar{\eta }$, the lower bound is exactly tight (${\bar{N}}_{W}/{\bar{N}}_{q}=1.0$) across the entire transmission range, and both curves overlap perfectly. The shaded region shows fluctuations due to sampling. (**b**) *Heterogeneous loss profile:* With 30% additive noise creating per-mode transmission variation (CV(η)∈[0.03,3.1]; mean CV(η)=67%), a clear gap emerges between ${\bar{N}}_{W}$ and ${\bar{N}}_{q}$. The exact ${\bar{N}}_{q}$ exceeds the lower bound ${\bar{N}}_{W}$ throughout, with median ratio ρ=0.86 and ranging from ρ≈0.84 at intermediate $\bar{\eta }$ to ρ≈0.95 at high $\bar{\eta }$.

A typical dependence of the complexity dimension on the mean transmission is presented in Figure 3. In the case of the homogeneous loss profile, the complexity dimension is given by the equal boson numbers ${\bar{N}}_{W}={\bar{N}}_{q}$, represented in Figure 3a by the single curve which exactly coincides with the analytical solution in Equation (18), averaged over the sweep of unitaries, as is explained above in Section 3.2.1. The variance in fluctuations, shown as a background strip marked in brown, increases with increasing transmission and boson numbers.

In the case of the heterogeneous loss profile when there is a finite loss variation, for example, with the transmission variation CV(η)∈[0.03,3.1] and mean CV(η)=67% as in Figure 3b, the above dependence splits into two curves—the lower bound given by Equation (7) and the exact boson number given by convex optimization in Equation (9)—represented by the lower and upper curves in Figure 3b: ${\bar{N}}_{W}^{\left(\sigma_{\eta }>0\right)}\left(\bar{\eta }\right)<{\bar{N}}_{W}^{\left(\sigma_{\eta }=0\right)}\left(\bar{\eta }\right)={\bar{N}}_{q}^{\left(\sigma_{\eta }=0\right)}\left(\bar{\eta }\right)<{\bar{N}}_{q}^{\left(\sigma_{\eta }>0\right)}\left(\bar{\eta }\right)$. Again, we conclude that the lower bound remains remarkably close to the exact value of the complexity dimension in the entire range of parameters. Note that in the limit of no losses, when $\bar{\eta }\to 1$, the lower bound $N_{W}$ tends to coincide with the exact value $N_{q}$. From Figure 3b and a large number of similar simulations for typical parameters of GBS experiments, we conclude that transmission heterogeneity is the necessary and sufficient condition for the lower bound looseness. The gap magnitude increases with heterogeneity level, providing experimental signatures for diagnosing loss non-uniformity in practical GBS devices.

**Figure 4:**
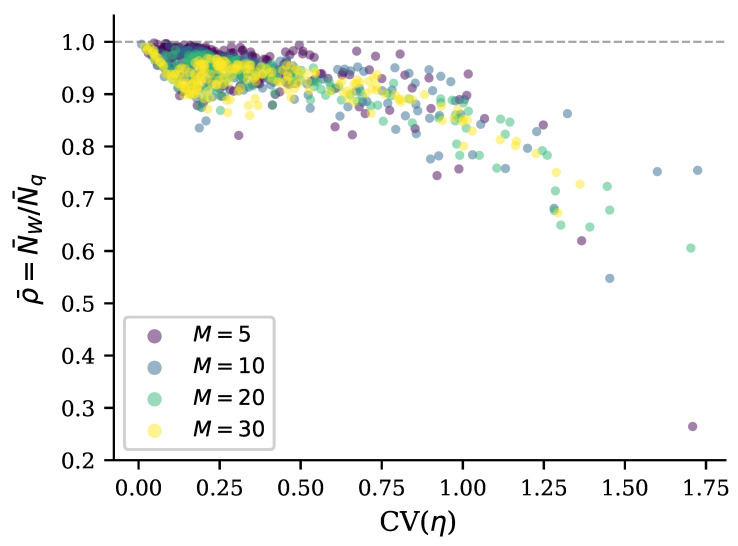
The ratio $\rho =N_{W}/N_{q}$ is a function of transmission heterogeneity CV(η) for systems with different numbers of modes M∈{5,10,20,30}. Each point represents a GBS instance with fixed mean squeezing $\bar{r}=0.8$ and varying mean transmission $\bar{\eta }$. The per-mode transmission values are drawn from a distribution centered at $\bar{\eta }$ with 15% additive noise, creating heterogeneity quantified by the coefficient of variation $\mathrm{CV}\left(\eta \right)=\sigma_{\eta }/\bar{\eta }$. The bound looseness (ρ<1) increases systematically with CV(η) regardless of mode count, demonstrating that the gap between $N_{W}$ and $N_{q}$ is governed by transmission heterogeneity rather than the system size. Points cluster along a common trajectory, indicating that the relationship between bound tightness and loss heterogeneity is scale-invariant. The horizontal dashed line at ρ=1 marks that perfect bound tightness is achieved only when CV(η)→0.

We now plot the ratio of boson numbers, $\rho =N_{W}/N_{q}$, in the case when we introduce mode-dependent losses by sampling per-mode transmissions with profile noise and then enforcing a target mean. Across these instances, as is shown in Figure 4, the tightness ρ significantly degrades as heterogeneity increases, that is, ρ decreases as CV(η) increases, with large variability in the fixed mean when CV(η) is large. Additionally, at a fixed heterogeneity level, lower mean transmission tends to yield looser bounds, consistent with the intuition that severe loss magnifies the disparity between the sub-vacuum and super-vacuum sectors of the covariance matrix spectrum.

Quantitatively, for the additive noise with variance $\sigma_{\eta }=0.15$, we find the median ρ≈0.934 (with a broad lower tail), while for the larger additive noise with variance $\sigma_{\eta }=0.30$, the median drops to ρ≈0.864. These values are computed over the full heterogeneous transmission sweeps.

**Figure 5:**
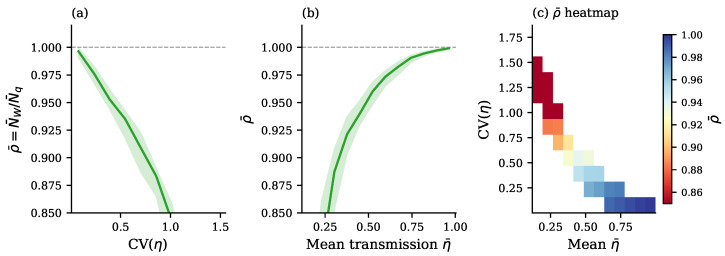
Detailed analysis of how loss heterogeneity affects Wigner lower bound tightness using multiplicative noise model (lognormal distribution preserving positivity). (**a**) $\bar{\rho }={\bar{N}}_{W}/{\bar{N}}_{q}$ versus CV(η): The bound tightens monotonically as heterogeneity decreases, approaching $\bar{\rho }=1$ as CV(η)→0. The shaded region indicates the interquartile range across instances sharing similar CV(η). (**b**) $\bar{\rho }$ versus mean transmission $\bar{\eta }$: At a fixed noise level, bound tightness shows weaker dependence on mean transmission than on heterogeneity, though slightly tighter bounds occur at intermediate $\bar{\eta }$ values. (**c**) Two-dimensional heatmap of median $\bar{\rho }$ across the $\left(\bar{\eta },\mathrm{CV}\left(\eta \right)\right)$ parameter space, revealing that CV(η) is the dominant predictor of bound looseness while $\bar{\eta }$ has secondary effects. The color scale (blue = tight; red = loose) confirms that decreasing CV(η) leads to tight bounds regardless of mean transmission level.

A detailed numerical analysis of the increasing deviation of the Wigner lower bound $N_{W}$ from the exact value of the complexity dimension $N_{q}$ with increasing loss variation CV(η) and loss mean $\bar{\eta }$ is presented in Figure 5. The dependence of the median ratio $\rho =N_{W}/N_{q}$, the closeness of which to unity characterizes the tightness of the Wigner lower bound, on both of the above parameters is colorfully presented by a heatmap in Figure 5c, which clearly demonstrates that the Wigner lower bound becomes loose only at a large enough loss variation, CV(η)>1, and very large losses (very small transmission η≪1).

### 3.4. Two Models of Loss Variability: Additive vs. Multiplicative Noise at Fixed Nominal Level

A central implementation detail is the precise noise model. Additive noise (Equation (34)) perturbs each mode by a Gaussian of a scale proportional to the allowed range, which, after clipping and mean enforcement, can still generate large CV(η), especially at low $\bar{\eta }$. Multiplicative noise (Equation (35)) applies a lognormal factor; after clipping and enforcement, it typically yields smaller induced CV(η) at the same $\sigma_{\eta }$. Empirically this leads to substantially tighter bounds: for multiplicative noise at $\sigma_{\eta }=0.15$, we find median ρ≈0.987, much closer to unity than the additive case.

### 3.5. Dependence of GBS Quantum Complexity on the Mode Squeezing: The Jensen Effect

**Figure 6:**
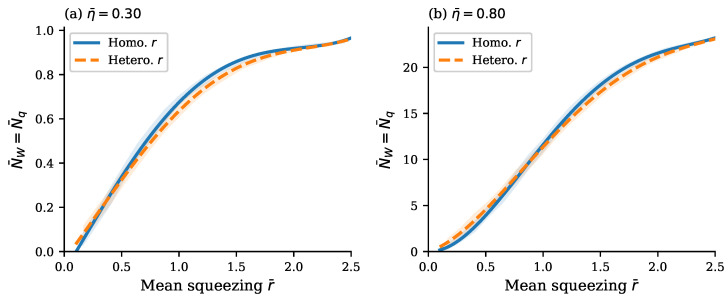
Squeezing heterogeneity does not affect bound tightness under homogeneous loss. (**a**) Low transmission ($\bar{\eta }=0.30$) and (**b**) high transmission ($\bar{\eta }=0.80$), each comparing homogeneous squeezing (blue solid) and heterogeneous squeezing with CV(r)≈47% (orange dashed). Shaded bands indicate fluctuations due to sampling within the 16th–84th percentile range. In both panels, ${\bar{N}}_{W}={\bar{N}}_{q}$ exactly ($\bar{\rho }=1.0000000001$), confirming that squeezing heterogeneity has no effect on the Wigner lower bound tightness when per-mode transmission is uniform. The magnitude difference between the curves is the Jensen effect arising due to the well-known Jensen inequality.

**Figure 7:**
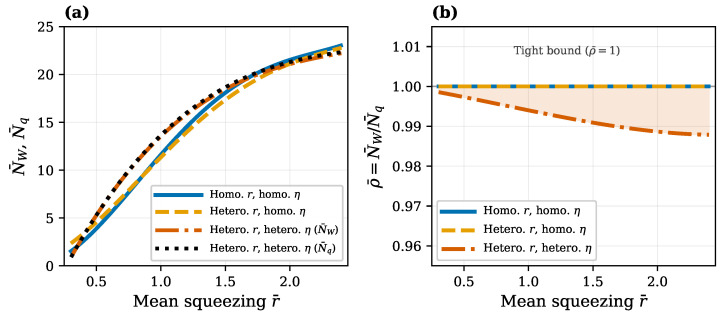
Three-way comparison of heterogeneous parameter effects on the Wigner lower bound. All three datasets have mean transmission $\bar{\eta }\approx 0.80$ and M=30 modes. (**a**) Wigner bound ${\bar{N}}_{W}$ and ${\bar{N}}_{q}$ versus mean squeezing $\bar{r}$. Homogeneous squeezing with homogeneous η (blue, solid), heterogeneous squeezing with homogeneous η (orange, dashed), and heterogeneous squeezing with heterogeneous η, showing both ${\bar{N}}_{W}$ (red-orange, dash–dot) and ${\bar{N}}_{q}$ (black, dotted). The difference between the homogeneous and heterogeneous squeezing curves at fixed homogeneous η arises from Jensen’s inequality. (**b**) The ratio $\bar{\rho }={\bar{N}}_{W}/{\bar{N}}_{q}$ versus mean squeezing. The homogeneous transmission cases overlap exactly at $\bar{\rho }=1$, demonstrating that squeezing heterogeneity alone does not affect bound tightness. Only the heterogeneous transmission case shows a deviation from unity, confirming that transmission heterogeneity is the sole driver of the Wigner lower bound looseness.

We now sweep the mean squeezing $\bar{r}$ while controlling the loss profile to test how the tightness of the Wigner lower bound $N_{W}$ depends on the overall squeezing strength and squeezing heterogeneity. For simplicity’s sake, we fix homogeneous loss at two representative operating points, $\bar{\eta }=0.3,0.8$ in Figure 6 and Figure 7, and sweep mean squeezing $\bar{r}$ using instance-mean stratified sampling to avoid bias toward intermediate means. In accordance with Section 3.2.1, boson numbers ${\bar{N}}_{W}$ and ${\bar{N}}_{q}$ are equal and increase monotonically with mean squeezing $\bar{r}$, consistent with the increased occupation ${\left[\eta \left(e^{-2r_{j}}-1\right)/2\right]}^{2}/\left[1+\eta \left(e^{-2r_{j}}-1\right)\right]$ of the quantum resource’s squeezed-vacuum modes, as per Equation (18).

However, the precise shape of this curve depends on the presence and the actual value of the squeezing variance, as is illustrated in Figure 6 and Figure 7. The blue curve represents the case of homogeneous squeezing, $\sigma_{r}=0$. The yellow dashed curve is plotted for heterogeneous squeezing. It yields larger boson numbers than the blue homogeneous-squeezing curve in the range of mean squeezing where the second derivative of the function $N_{W}\left(\bar{r}\right)$ is positive, since large values of squeezing within the variance interval dominate because the function $N_{W}\left(\bar{r}\right)$ is convex. In the range of mean squeezing where the second derivative of the function $N_{W}\left(\bar{r}\right)$ is negative, the situation is opposite: the function $N_{W}\left(\bar{r}\right)$ is concave and the yellow dashed heterogeneous-squeezing curve goes below the green homogeneous-squeezing curve. This is *the Jensen effect* since such an inequality for a concave function f(r) of a random variable *r* is known as Jensen’s inequality (symbol E denotes averaging)

(37) E[f(r)]≤f(E[r]).

Overall, the effect of squeezing heterogeneity on the quantum complexity is the second-order effect (since it is due to the second derivative) and, hence, it is relatively weak compared to the effect of loss heterogeneity. Figure 6 and Figure 7 confirm this conclusion.

At last, let us look at the combined effect of the loss heterogeneity and squeezing heterogeneity illustrated in Figure 7 for the case of a relatively high mean transmission $\bar{\eta }=0.8$ in the system of M=30 modes. In this case, the loss heterogeneity makes boson numbers ${\bar{N}}_{W}$ and ${\bar{N}}_{q}$ unequal and, contrary to the two other cases shown in Figure 7a by blue and yellow curves, they are represented now by two different orange and black dotted curves, respectively. However, the ${\bar{N}}_{W}$ curve is located very close to (just a bit below) the ${\bar{N}}_{q}$ curve, since their ratio $\bar{\rho }={\bar{N}}_{W}/{\bar{N}}_{q}$ is less than unity only by about 1%, as is justified in Figure 7b. This is because the mean transmission $\bar{\eta }=0.8$ is not much less than the unity that would be necessary for the appearance of a large deviation in the Wigner lower bound from the exact value ${\bar{N}}_{q}$, as per the above discussion of the heatmap in Figure 5c. Another interesting observation from Figure 7a is that the loss heterogeneity increases quantum computational complexity but not by much and only for intermediate mean squeezing $\bar{r}\in \left(0.5,1.5\right)$.

**Figure 8:**
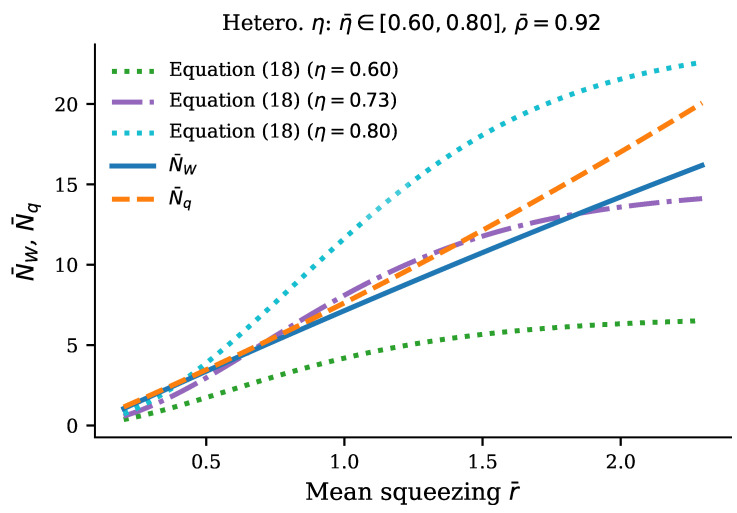
Effect of transmission heterogeneity on boson number bounds as functions of squeezing. The figure shows the Wigner lower bound ${\bar{N}}_{W}$ (blue solid) and exact quantum resource ${\bar{N}}_{q}$ (orange dashed) versus the mean squeezing for M=30 mode GBS instances with heterogeneous per-mode transmission, filtered to $\bar{\eta }\in \left[0.60,0.80\right]$ (349 instances). Three analytic Equation (18) reference curves show $N_{W}$ for systems with *a homogeneous transmission profile* at η=0.60, 0.73, and 0.80. The visible gap between the Wigner lower bound ${\bar{N}}_{W}$ and the exact value ${\bar{N}}_{q}$ (median $\bar{\rho }=0.92$) appear due to transmission heterogeneity, confirming that the transmission heterogeneity—not the mean transmission level—causes the Wigner lower bound to become loose. The cone of the boson numbers $N_{W}=N_{q}$, swept by the analytic solutions in Equation (18) for transmissions within the range of transmission heterogeneity $\bar{\eta }\in \left[0.60,0.80\right]$, is indicated by the green and blue dotted curves. As expected, the split curves of boson numbers $N_{W}$ and $N_{q}$ both lie close to the central solution of Equation (18) for $\bar{\eta }\approx 0.73$ (violet) within the above cone.

Finally, the aforementioned effect of transmission heterogeneity on the splitting of the Wigner lower bound $N_{W}$ from the exact boson number in the quantum resource $N_{q}$ as the function of squeezing is disclosed in Figure 8 in a more pronounced form due to the smaller transmission and larger variation in squeezing. Its description and comparison against analytic solutions in Equation (18) are given in the figure caption.

### 3.6. Dependence of GBS Quantum Complexity on the Number of Modes

We next study scaling with the number of modes *M* in both homogeneous and heterogeneous loss settings. In all cases we keep the mean squeezing and mean loss fixed, so that increasing *M* increases the number of available squeezed resources while preserving the per-mode operating point.

For the homogeneous baseline, we fix $\bar{r}=0.8$ and homogeneous power transmission $\bar{\eta }=0.3$ with no profile noise, and sweep M∈{5,10,20,30}. In accordance with Section 3.2.1, boson numbers $N_{W}$ and $N_{q}$ are equal and increase with the number of modes *M*, as expected from the additivity of boson numbers across modes since the sum over modes is presented in Equation (18).

The case of heterogeneous losses for different numbers of modes is presented in Figure 4. We introduce mode-dependent loss by adding profile noise to the power transmission profile while controlling the mean. Specifically, we sample per-mode power transmissions with target mean $\bar{\eta }=0.3$, apply either additive or multiplicative noise (Equations (34) and (35)) in the power-transmission metric, and enforce the target mean. In this regime, the ratio of the lower bound to the exact boson number in the quantum resource, $\rho =N_{W}/N_{q}$, drops below unity and correlates strongly with the induced heterogeneity CV(η). Importantly, once CV(η) is accounted for, the dependence of the ratio $\rho =N_{W}/N_{q}$ on the number of modes *M* is comparatively weak over the tested range, suggesting that the dominant mechanism controlling tightness of the lower bound is the *distributional spread* of loss rather than mode count itself. The point is that, although the two boson numbers are not equal in the case of the heterogeneous loss profile, $N_{W}\neq N_{q}$, both of them grow proportionally to the number of modes if *M* is large enough that various losses and squeezings are uniformly distributed across the modes.

### 3.7. Spectrum of Eigenvalues and Spectral Asymmetry of the Covariance Matrix

**Figure 9:**
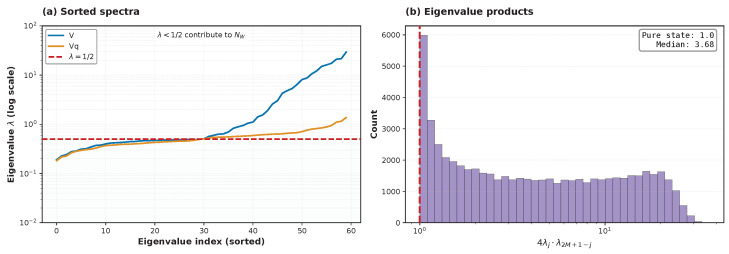
(**a**) Spectrum of eigenvalues enumerated in ascending order for the full output covariance matrix *V* (blue) and for its quantum resource’s part $V_{q}$ (yellow) at the exit from a lossy interferometer. (**b**) Spectral spreading in the distribution of the pairing products, $4\lambda_{j}\lambda_{2M+1-j},j=1,\ldots ,M=30$, of the eigenvalues (enumerated in ascending order) of the output covariance matrix *V* after passing through a lossy interferometer. The input covariance matrix of bosons in the pure squeezed-vacuum states, Equations (23) and (24), has all pairing products perfectly concentrated at unity, $4\lambda_{j}^{\left(in\right)}\lambda_{2M+1-j}^{\left(in\right)}\equiv 1$.

To connect quantum complexity and its lower bound trends to the geometrical structure of the Wigner quasiprobability iso-density ellipsoids, we inspect the ordinary eigenvalue spectrum $\left\{\lambda_{j}\right\}$ of the quadrature covariance matrix *V* shown in Figure 9a. Because the Wigner lower bound $N_{W}$ is constructed solely from the sub-vacuum part of this spectrum via Equations (6) and (7), the distribution of eigenvalues relative to $\lambda_{\mathrm{vac}}=1/2$ directly controls the lower bound for the number of squeezed-vacuum bosons in the quantum resource. Counting sub-vacuum eigenvector directions in a diagnostic ensemble of per-mode uniform instances, we observe that exactly *M* eigenvalues lie below 1/2 across all tested samples, as can be seen in Figure 9a. This is consistent with the fact that our states are generated from *M* squeezed-vacuum inputs followed by passive mixing and loss channels: sub-vacuum fluctuations exist, but are limited in number by the available squeezed resources.

#### 3.7.1. Asymmetry Between Sub-Vacuum, λ<1/2, and Super-Vacuum, λ>1/2, Sectors

**Figure 10:**
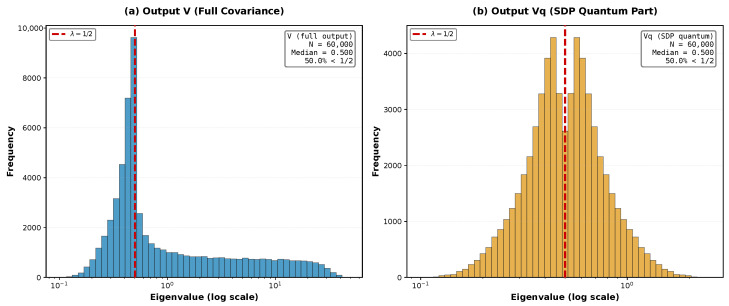
Eigenvalue distribution for (**a**) the full output covariance matrix *V* and (**b**) its quantum resource’s part, $V_{q}$. Note that a long super-vacuum tail of large eigenvalues $\lambda_{j}\gg 1/2$ associated with classical bosons appeared due to dissipative decay of the input pure quantum squeezed bosons.

The pooled eigenvalue distribution for the output covariance matrix shows a sharp accumulation near (and below) $\lambda_{\mathrm{vac}}=1/2$ together with a long super-vacuum tail, as is shown in Figure 10a. This asymmetry explains why heterogeneous loss can loosen the Wigner lower bound: loss and loss heterogeneity preferentially inflate super-vacuum directions in the Wigner quasiprobability distribution with classical bosons, but they cannot create new squeezed bosons; they can only remove them from the sub-vacuum directions, reducing contributions to the Wigner lower bound of the boson number in the quantum resource $N_{W}$, as shown in Equation (7). At the same time, the eigenvalue distribution of the output quantum resource’s covariance matrix $V_{q}$ is perfectly symmetrical relative to the quantum vacuum level $\lambda_{\mathrm{vac}}=1/2$ with two symmetrically located maxima, as is shown in Figure 10b.

#### 3.7.2. The Smallest–Largest Pairing of Eigenvalues: Squeezed Bosons vs. Classical Bosons

For a single mode in a diagonal basis, the two quadrature variances multiply to 1/4 in the pure squeezed-vacuum state. However, for general multimode lossy states, products of *ordinary* eigenvalues $4\lambda_{j}\lambda_{2M+1-j}$ are not symplectic invariants and need not concentrate at unity. Hence, we can use a spectral spread in the distribution of the pairing products, exemplified in Figure 9b, as a qualitative diagnostic of the presence of classical bosons and the degree of their excess over squeezed bosons. The large counts and wide spread of pair-wise products $4\lambda_{j}\lambda_{2M+1-j},j=1,\ldots ,M$, to the right from unity in Figure 9b, clearly tell us that there is a large excess of classical bosons over the pure squeezed-vacuum bosons in most of the modes due to their generation of classical bosons in the dissipative process of losses.

A fully symplectic diagnostic would instead examine symplectic eigenvalues $\nu_{k}$ of *V* (all satisfying $\nu_{k}\geq 1/2$) and so this calls for comparison of the squeezed modes of quantum resources against quasiparticles and eigen-squeezed modes arising in the Bloch-Messiah or Williamson decomposition of the covariance matrix. We leave such an analysis for future refinement.

## 4. Upper Bound for the Complexity Dimension of the Quantum Resource

The upper bound for the complexity dimension of the quantum resource can be introduced similarly to the lower one (see Section 2.1) if, instead of the covariance matrix’s eigenvalues being less than 1/2, $\left\{\lambda_{j}^{\left(-\right)}<1/2|j=1,\ldots ,K\right\}$, we choose the eigenvalues that are larger than 1/2, $\left\{\lambda_{j}^{\left(+\right)}>1/2|j=2M-K^{\left(+\right)},\ldots ,2M\right\}$, for building the squeezed-vacuum modes of the quantum resource’s part of the covariance matrix *V*. Then, instead of Equations (6) and (7), we set

(38) $$\lambda_{j}^{\left(+\right)}=\frac{e^{2r_{j}}}{2},\;n_{j}=\sinh^{2}r_{j}=\frac{1}{4}\left(2\lambda_{j}^{\left(+\right)}-2+\frac{1}{2\lambda_{j}^{\left(+\right)}}\right)\;\forall j\geq \max\left\{M,2M-K^{\left(+\right)}\right\},$$

(39) $$N_{W}^{\left(+\right)}=\sum_{j=2M-K^{\prime }}^{2M}\sinh^{2}r_{j}=\frac{1}{4}\sum_{j=2M-K^{\prime }}^{2M}\left(2\lambda_{j}^{\left(+\right)}-2+\frac{1}{2\lambda_{j}^{\left(+\right)}}\right),\;K^{\prime }=\min\left\{M,K^{\left(+\right)}\right\}.$$

The Loewner order’s implication in Equation (20) for these eigenvalues remains valid. However, the sign in front of the squeezing parameter in the exponent for these eigenvalues is now different (positive) both for $\mu_{j}=\frac{1}{2}e^{2r_{j}^{\left(q\right)}}$ and $\lambda_{j}^{\left(+\right)}=\frac{1}{2}e^{2r_{j}},j=2M-K^{\prime },\ldots ,2M$ (compare Equations (6) and (38)). This amounts to reversing the sign of inequalities in all steps of the lower-bound proof in Equation (22), and it means now that the boson number in Equation (39) is the upper bound for the computational complexity dimension of the quantum resource:

(40) $$r_{j}\geq r_{j}^{\left(q\right)}\;\Rightarrow \;n_{j}=\sinh^{2}r_{j}\geq n_{j}^{\left(q\right)}=\sinh^{2}r_{j}^{\left(q\right)}\;\forall j=2M-K^{\prime },\ldots ,2M\;\Rightarrow \;N_{W}^{\left(+\right)}\geq N_{q}.$$

However, the physics behind the lower and upper bounds is different. The lower bound in Equation (7) is the number $N_{W}$ of squeezed bosons determined by the covariance matrix’s eigenvalues, $\left\{\lambda_{j}^{\left(-\right)}<1/2|j=1,\ldots ,K\right\}$, below the threshold of nonclassicality $\lambda_{\mathrm{vac}}=1/2$, and originates due to the intrinsically quantum effects of the noncommutativity of the coordinate and momentum operators and Heisenberg’s uncertainty. This number $N_{W}$ is strongly limited by the amount of squeezing that is originally introduced or created in the system via input squeezed bosons. It cannot increase during propagation in a lossy interferometer.

On the contrary, the upper bound in Equation (39), $N_{W}^{\left(+\right)}$, incorporates all classical bosons which appear due to losses, both thermal and other sources of classical noise, including propagation along lossy channels. The number $N_{W}^{\left(+\right)}$ and the covariance matrix’s eigenvalues which determine it, $\left\{\lambda_{j}^{\left(+\right)}>1/2|j=2M-K^{\prime },\ldots ,2M\right\}$, are not limited by quantum constraints, could be arbitrarily large, and greatly grow with the system’s scaling. As a result, the upper bound in Equation (39) is usually more characteristic of the classical contents of the boson system rather than of its quantum resource. So, the upper bound number $N_{W}^{\left(+\right)}$ could be many times larger than the exact number of squeezed-vacuum bosons in the quantum resource and, contrary to the quite accurate lower bound in Equation (7), is not very relevant to the quantum resource for computational complexity.

This conclusion can be viewed as a manifestation of the fundamental quantum-classical asymmetry in the properties of many-body systems’ fluctuations in general and in the behavior of the covariance matrix’s eigenvalues in particular.

## 5. Conclusions

We conclude that the quantum computational complexity of Gaussian boson sampling and, more generally, the quantum number statistics of many-body boson systems in Gaussian states are closely related to geometrical squeezing of multi-dimensional Wigner quasiprobability iso-density ellipsoids below a nonclassical, quantum-vacuum threshold level. Amazingly, calculation of the quantum complexity dimension by simply finding the minor axes of these Wigner ellipsoids via standard diagonalization methods and using the explicit formula in Equation (7), as proposed in this work, gives a reliable easy-to-compute Wigner lower bound for the complexity dimension which is quite close to the exact value computed via numerical convex optimization according to the best of all known classical algorithms. Numerical analysis shows that in a wide range of parameters, typical for known experiments, the relative deviation of the Wigner lower bound from the exact value on average is less than about 20% (Figure 1 and Figure 2).

In proposing and building the current and future experiments on Gaussian boson sampling, researchers should start with analysis of the ground-truth covariance matrix (see, for example, discussion in [9,21,22,29]). The above fact, together with the other remarkable properties of the estimate in Equation (7) and the rigorous proof that Equation (7) yields the exact lower bound, makes the proposed Wigner lower bound in Equation (7) a reliable, practical, and rigorous tool for designing setups of guaranteed high computational complexity. Importantly, this is true not just for the experiments on Gaussian boson sampling, but also for other quantum advantage experiments in many-body boson systems in Gaussian states, since the proposed estimate of quantum computational complexity relies on the geometrical complexity of Wigner distribution in Equation (1), which fully describes and incorporates all quantum statistical properties of such systems. Note that revealing the origin of the computational complexity of quantum computing in general, including universal quantum computing with multi-qubit systems; circuit models to quantum simulations; one-way quantum computing; and various nonequilibrium, non-Markovian, non-bosonic, and other systems whose quantum state is not fully described by the covariance matrix, requires much wider analysis.

Analytical and numerical results presented above reveal the origin of bosons constituting the system’s quantum computational resource responsible for the ♯P-hard computational complexity, that is, quantum advantage over classical computers. Namely, those are the squeezed-vacuum bosons which have a significant overlap with the superposition of coordinate and momentum operators aligned in the phase space along one of the strongly squeezed minor axes of the Wigner quasiprobability iso-density ellipsoids. This conclusion provides an intuitively appealing and physically transparent picture of the actual contents of the quantum computational resource which, in general, is different from the commonly discussed quasiparticles or eigen-squeezed modes of the many-body boson system.

The above results present a clear qualitative and quantitative picture of how quantum computational complexity grows with increasing transmission of the interferometer (Figure 3 and Figure 5) and increasing squeezing of the input bosons (Figure 6, Figure 7 and Figure 8). Remarkably, near the maximum complexity, the established lower bound in Equation (7) tends to coincide with the exact complexity dimension computed by convex optimization (Figure 3a and Figure 6). The main factor that both degrades complexity and decreases accuracy of the estimate in Equation (7) is heterogeneity of losses across modes, quantified by the coefficient of transmission variation in Equation (33) (see Figure 4, Figure 5, Figure 7, and Figure 8). Variation in squeezing across modes reveals some interesting effects, such as the Jensen effect described in Section 3.5, but does not significantly affect complexity and accuracy of the Wigner lower bound (7), as is seen in Figure 6, Figure 7 and Figure 8. Increasing the number of modes by adding more modes with parameters close to their mean values over the set of modes increases complexity proportionally to the number of modes but does not change the accuracy of the lower bound much (see Figure 4).

Very promising for further analysis of quantum advantage are the remarkable manifestations of improvement or degradation in quantum computational complexity in the evolution of the spectrum of the covariance matrix due to variation in transmission, squeezing, and other parameters of the multimode boson system described in Section 3.7. These manifestations include, for example, the appearance of a long super-vacuum tail in the spectrum, asymmetry relative to the vacuum threshold, and the large counts and wide spread of the smallest–largest pair-wise products of eigenvalues presented in Figure 9 and Figure 10.

The proposed approach based on the geometry of Wigner distribution looks very fruitful for disclosing the mysteries of quantum computational advantage and formulating related open questions. For instance, a comparative analysis of the dependence of spectra of the system’s covariance matrix and its quantum resources and classical parts on losses and squeezing as well as on their variations across modes suggests very interesting correlations and open problems for the formation of the quantum computational resource and quantum advantage (see Figure 9 and Figure 10). Here we name just a few of such open problems:

(i) Finding a procedure for the explicit analytical construction of the quantum resource’s modes, starting from the eigenvectors of the covariance matrix by constructing their conjugated pair’s counterparts via the symplectic Gram–Schmidt procedure [30] applied to the sub-vacuum, nonclassical sector of the eigenvectors ordered in ascending order of their eigenvalues, $\lambda_{j}<\lambda_{\mathrm{vac}}=1/2,j=1,\ldots ,K$, as per Equation (10).
(ii) Comparison of the squeezed modes of the quantum complexity resource against quasiparticles and eigen-squeezed modes arising in the Bloch-Messiah or Williamson decomposition of the covariance matrix.
(iii) Finding a protocol for constructing fully optimized classical part $V_{c}$ of the covariance matrix decomposition in Equation (8) via consecutive nullification of all covariance matrix eigenvalues related to the sub-vacuum, nonclassical sector by moving an appropriate part of the covariance matrix *V* into the quantum resource’s part $V_{q}$.

Resolution of such open problems, especially by means of analytical, algebraic, and combinatorial tools, promises novel important insights into the nature of quantum advantage. Thus, the proposed approach certainly requires further development.

## Data Availability

The data that support the findings of this study are available from the corresponding author upon reasonable request.
